# Cribriform architecture in radical prostatectomies predicts oncological outcome in Gleason score 8 prostate cancer patients

**DOI:** 10.1038/s41379-020-0625-x

**Published:** 2020-07-20

**Authors:** Eva Hollemans, Esther I. Verhoef, Chris H. Bangma, John Rietbergen, Susanne Osanto, Rob C. M. Pelger, Tom van Wezel, Henk van der Poel, Elise Bekers, Jozien Helleman, Monique J. Roobol, Geert J. L. H. van Leenders

**Affiliations:** 1 Department of Pathology, Erasmus MC, University Medical Center, Rotterdam, The Netherlands; 2 Department of Urology, Erasmus MC, University Medical Center, Rotterdam, The Netherlands; 3 Department of Urology, Franciscus Gasthuis & Vlietland, Rotterdam, The Netherlands; 4 Department of Medical Oncology, Leiden University Medical Center, Leiden, The Netherlands; 5 Department of Urology, Leiden University Medical Center, Leiden, The Netherlands; 6 Department of Pathology, Leiden University Medical Center, Leiden, The Netherlands; 7 Department of Urology, Netherlands Cancer Institute, Amsterdam, The Netherlands; 8 Department of Pathology, Netherlands Cancer Institute, Amsterdam, The Netherlands

**Keywords:** Prostate cancer, Prostate cancer

## Abstract

The Gleason score is an important parameter for clinical outcome in prostate cancer patients. Gleason score 8 is a heterogeneous disease including Gleason score 3 + 5, 4 + 4, and 5 + 3 tumors, and encompasses a broad range of tumor growth patterns. Our objective was to characterize individual growth patterns and identify prognostic parameters in Gleason score 8 prostate cancer patients. We reviewed 1064 radical prostatectomy specimens, recorded individual Gleason 4 and 5 growth patterns as well as presence of intraductal carcinoma, and evaluated biochemical recurrence- and metastasis-free survival. Gleason score 8 disease was identified in 140 (13%) patients, of whom 76 (54%) had Gleason score 3 + 5, 46 (33%) 4 + 4, and 18 (13%) 5 + 3 disease. Invasive cribriform and/or intraductal carcinoma (*n* = 87, 62%) was observed more frequently in Gleason score 4 + 4 (93%) than 3 + 5 (47%; *P* < 0.001) and 5 + 3 (44%; *P* < 0.001) patients. Gleason pattern 5 was present in 110 (79%) men: as single cells and/or cords in 99 (90%) and solid fields in 32 (29%) cases. Solid field pattern 5 coexisted with cribriform architecture (23/32, 72%) more frequently than nonsolid pattern 5 cases (36/78, 46%, *P* = 0.02). In multivariable analysis including age, prostate-specific antigen, pT-stage, surgical margin status, and lymph node metastases, presence of cribriform architecture was an independent parameter for biochemical recurrence-free (hazard ratio (HR) 2.0, 95% confidence interval (CI) 1.0–3.7; *P* = 0.04) and metastasis-free (HR 3.5, 95% CI 1.0–12.3; *P* = 0.05) survival. In conclusion, invasive cribriform and/or intraductal carcinoma occurs more frequently in Gleason score 4 + 4 prostate cancer patients than in Gleason score 3 + 5 and 5 + 3, and is an independent parameter for biochemical recurrence and metastasis. Therefore, cribriform architecture has added value in risk stratification of Gleason score 8 prostate cancer patients.

## Introduction

The Gleason grading system for prostate cancer is based on classification of histomorphological growth patterns [1]. At the 2014 meeting of the International Society of Urological Pathology (ISUP), consent was reached that a Grade Group should be reported in conjunction with the Gleason score, based on the initial work of Pierorazio et al. which was endorsed by the World Health Organization (WHO) in 2016 [2–4]. The Grade Group system is comprehensive and facilitates patient communication as it labels Gleason score 2–6 as Grade Group 1 and emphasizes the important distinction between Gleason score 3 + 4 = 7 (Grade Group 2) and 4 + 3 = 7 (Grade Group 3) prostate cancer. Grade Group 4 prostate cancer encompasses Gleason score 8 tumors, including Gleason score 3 + 5, 5 + 3, and 4 + 4 [5, 6]. However, it is not yet clear whether these three Gleason score 8 subgroups have similar clinical outcome.

The importance of distinguishing individual prostate cancer growth patterns is increasingly being acknowledged. Gleason pattern 4 encompasses four major growth patterns, including poorly formed, fused, glomeruloid and cribriform glands [3]. The clinical relevance of cribriform architecture in prostate cancer has been well established in recent years, as it is associated with biochemical recurrence, metastasis, and disease-specific death [7–13]. Intraductal carcinoma is characterized by a proliferation of malignant epithelial cells with cribriform or solid architecture distending preexistent acini and prostatic ducts with preservation of basal cells [3]. Although not incorporated in the Gleason score or Grade Group, intraductal carcinoma is independently associated with adverse oncological outcome [8, 14, 15]. The adverse impact of invasive cribriform and intraductal carcinoma has mainly been studied in Gleason score 3 + 4 prostate cancer, as it might affect clinical decision-making in this patient population in particular. Some studies indicate that presence of invasive cribriform and intraductal carcinoma also has independent predictive value in Gleason score 8 prostate cancer patients [14, 16].

While the impact of cribriform architecture is well recognized, little is known about the clinical relevance of individual Gleason 5 growth patterns [7]. Gleason pattern 5 encompasses tumor growth in single cells, cords, and solid fields [3]. Furthermore, presence of comedonecrosis is considered Gleason pattern 5, whether it is present within papillary, cribriform, or solid fields. Of notice, recent studies have shown that comedonecrosis more commonly occurs in intraductal carcinoma than in invasive carcinoma, requiring basal cell immunohistochemistry for their distinction [17–19]. While Gleason score 8 prostate cancer is generally considered a high-risk disease requiring immediate therapeutic intervention, analysis of individual Gleason 4 and 5 growth patterns might attribute to risk stratification and optimize personalized treatment decisions. The objective of this study is to compare the clinical characteristics and outcome of Gleason score 3 + 5, 5 + 3, and 4 + 4 subgroups and to investigate the impact of invasive cribriform and/or intraductal carcinoma in Gleason score 8 radical prostatectomy specimens.

## Methods

### Patient selection

Patients who had undergone radical prostatectomy for prostatic adenocarcinoma from three university medical centers in The Netherlands between 2000 and 2017 were included in this study; 854 patients were operated at Erasmus MC, University Medical Center, Rotterdam; 96 at Leiden University Medical Center (LUMC), Leiden; and 137 at Antoni van Leeuwenhoek Hospital, the Netherlands Cancer Institute (NKI), Amsterdam. Whereas the radical prostatectomies from Erasmus MC were consecutive, those from LUMC and NKI were selected for presence of Gleason score 4 + 3 to 10 in the original pathology report. We excluded men who had undergone hormonal, radiation, and/or viral therapy (*n* = 23) prior to operation [20]. Radical prostatectomy specimens were fixed in neutral-buffered formalin, after which they were sectioned transversely and embedded entirely for diagnostic purposes. All slides were available for pathology review. This study was approved by the institutional Medical Research Ethics Committee (MEC-2018-1614).

### Pathologic evaluation

All 1064 radical prostatectomy specimens were reviewed in common sessions by two investigators (EH, GvL), blinded to clinical outcome. For each specimen the following features were recorded: Gleason score and Grade Group according to the 2014 ISUP/2016 WHO guidelines, pT-stage according to the American Joint Committee on Cancer TNM 8th edition, surgical margin status, presence of intraductal carcinoma, and percent Gleason 4 and 5 growth patterns [3, 21]. In case of multifocality we only monitored the characteristics of the index tumor defined as the tumor with the highest grade, stage or volume.

**Fig. 1:**
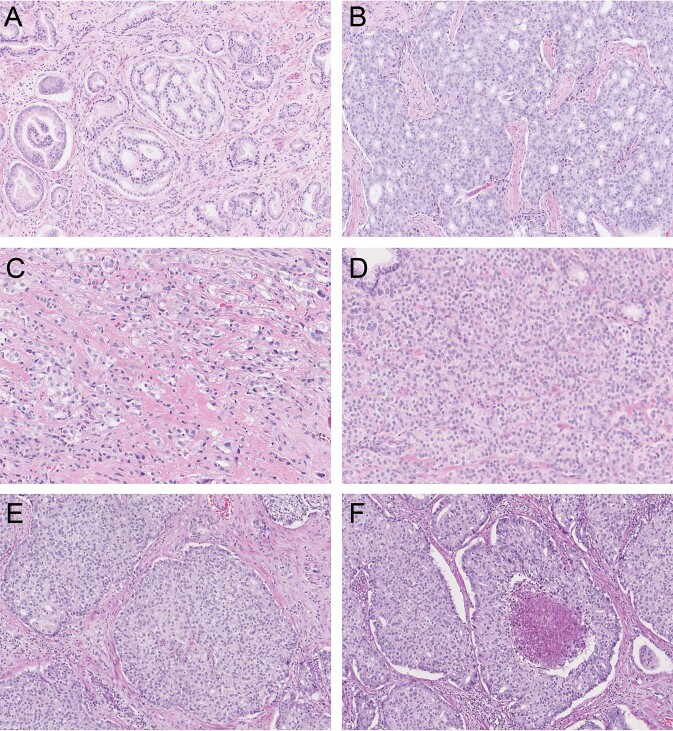
Gleason pattern 4 and pattern 5 tumor morphology. **a** Gleason pattern 4, small invasive cribriform structures, 15×. **b** Gleason pattern 4, large invasive cribriform structures, 10×. **c** Gleason pattern 5, cords, 20×. **d** Gleason pattern 5, small solid nests with subtle intervening stroma, 20×. **e** Gleason pattern 5, medium to large sized solid fields, 15×. **f** Gleason pattern 5, comedonecrosis in a solid field, 15×.

The following Gleason 4 growth patterns were recognized: poorly formed, fused, glomeruloid, and cribriform glands [2, 3]. Furthermore, we distinguished small and large cribriform gland architecture (Fig. 1a, b), since the latter is associated with more aggressive behavior [12]. Large cribriform structures were defined as having a diameter more than twice the size of adjacent benign glands. We examined the following Gleason 5 growth patterns: single cells, cords, and solid fields (Fig. 1c–f). Single cells and cords were grouped for analysis. Solid fields were divided into those with small solid nests containing 10–30 cells, and those consisting of medium to large solid fields with more than 30 cells. In case comedonecrosis was present in invasive cribriform or solid fields, this was considered Gleason pattern 5. Invasive cribriform Gleason pattern 4 and solid pattern 5 either with or without comedonecrosis were morphologically distinguished from intraductal carcinoma based on the following features: invasive cribriform and solid prostate cancer had irregular borders or formed interconnecting fields, well exceeding the outline of distended preexistent glands, or extended into periprostatic adipose tissue, ejaculatory ducts or seminal vesicles. Intraductal carcinoma was continuous with preexistent glands lined by basal cells, or contained corpora amylacea. In case invasive cribriform or solid carcinoma and intraductal carcinoma could not be differentiated by morphological criteria alone, additional basal cell immunohistochemistry was performed. Basal cell immunohistochemistry (34BE12) was performed in 189/854 (22%) radical prostatectomy specimens from Erasmus MC, including 14/31 (45%) Gleason score 8 tumors with cribriform or solid architecture; no paraffin blocks were available from the other hospitals. If basal cells were completely absent, the lesion was classified as either invasive cribriform Gleason pattern 4 or solid pattern 5 carcinoma. When sporadic, scattered or continuous basal cells were identified, the lesion was considered intraductal carcinoma. Intraductal carcinoma and tertiary patterns were not incorporated in the Gleason score [2, 3, 22]. Minor high-grade components occupying <5% of the tumor volume were considered as tertiary pattern. The Grade Group concordance rate at revision was 88/135 (65%) for radical prostatectomies from NKI and 39/94 (41%) for specimens from LUMC.

### Clinical follow-up

Clinical follow-up after radical prostatectomy consisted of 6 monthly, and later annual monitoring of serum prostate-specific antigen (PSA) levels. Biochemical recurrence was defined as PSA levels ≥0.2 ng/ml measured at two consecutive points in time, at least 3 months apart with undetectable PSA levels after operation, or as PSA increase of >2.0 ng/ml when serum PSA had not declined to zero after operation. Postoperative lymph node and distant metastases were confirmed by biopsy or multidisciplinary consensus.

### Statistical analysis

Continuous variables with normal distribution were analyzed using the independent sample Student’s *t* test for two groups, or one-way ANOVA for ≥3 groups. Variables without normal distribution were analyzed using the Mann–Whitney *U* test for two groups, or Kruskal–Wallis test for ≥3 groups. For comparison of categorical parameters Pearson’s chi squared (*χ*^2^) test was used, and Fisher’s exact test in case of small numbers (*n* ≤ 20). Missing PSA values (*n* = 27) were imputed using the median PSA value. Biochemical recurrence-free survival and metastasis-free survival were analyzed using Cox proportional hazards model and visualized by Kaplan–Meier curves. Statistics were performed using SPSS version 24 (IBM, Chicago, IL, USA). Results were considered significant when the two-sided *P* value was <0.05.

## Results

### Characteristics of Gleason score 8 prostate cancer patients

Out of 1064 radical prostatectomy specimens, 140 (13%) had Gleason score 8 prostate cancer. The median age of Gleason score 8 patients was 65.3 years (interquartile range (IQR) 61.4–68.5 years) and median serum PSA level was 10.0 ng/ml (IQR 7.2–16.0 ng/ml). Gleason scores were distributed as follows: 76 (54%) men had Gleason score 3 + 5, 46 (33%) Gleason score 4 + 4, and 18 (13%) Gleason score 5 + 3. Pathologic tumor stage was T2 in 67 (48%) men, T3a in 44 (31%), and T3b in 28 (20%). One (1%) patient had a T4 tumor and was grouped with T3b tumors for further analysis. Positive surgical margins were present in 68 (49%) cases. Pelvic lymph node dissection was performed in 91 (65%) men, 12 (9%) of whom had lymph node metastasis. Median follow-up time was 68.7 months (IQR 36.7–102.8).

### Clinicopathological features and outcome of Gleason score 3 + 5, 4 + 4, and 5 + 3

The clinicopathological features of Gleason score 8 patients stratified for Gleason score are shown in Table 1. The median PSA level of patients with Gleason score 5 + 3 prostate cancer was 13.4 ng/ml (IQR 8.8–26.8 ng/ml), significantly higher than for men with Gleason score 3 + 5 (10.0 ng/ml; IQR 7.4–15.0 ng/ml; *P* = 0.05) and Gleason score 4 + 4 (8.9 ng/ml; IQR 6.9–16.0 ng/ml; *P* = 0.03). PSA levels of Gleason score 3 + 5 and 4 + 4 were comparable (*P* = 0.45). Age, pT-stage, surgical margin status and lymph node metastases were not significantly different between groups. While Gleason pattern 4 constituted ≥95% of the tumor volume in Gleason score 4 + 4 by definition, it was present in 73/76 (96%) Gleason score 3 + 5 and 12/18 (67%) 5 + 3 tumors. The median percentage of Gleason pattern 4 was 30% (IQR 20–35%) in 3 + 5 tumors and 18% (IQR 0–21%) in 5 + 3 tumors (*P* < 0.001). Tertiary (<5%) Gleason pattern 5 was observed in 16/46 (35%) Gleason score 4 + 4 tumors.

**Table 1:** Gleason score 8 patients stratified for individual Gleason score (GS).

	All *n* = 140	GS 3 + 5 *n* = 76	GS 4 + 4 *n* = 46	GS 5 + 3 *n* = 18	*P* value
Age (years)	64.7 (65.3; 61.4–68.5)	64.3 (64.5; 60.7–68.2)	65.6 (66.2; 62.4–69.2)	64.5 (65.2; 60.4–67.2)	0.46
PSA (ng/ml)	12.9 (10.0; 7.2–16.0)	12.5 (10.0; 7.4–15.0)	11.5 (8.9; 6.9–16.0)	18.5 (13.4; 8.8–26.8)	0.07
pT-stage					
T2	67 (48%)	41 (54%)	19 (41%)	7 (39%)	0.35
T3a	44 (31%)	19 (25%)	19 (41%)	6 (33%)	
T3b/T4	29 (21%)	16 (21%)	8 (18%)	5 (28%)	
Overall cribriform	87 (62%)	36 (47%)	43 (93%)	8 (44%)	<0.001
Gleason pattern 4					
Small cribriform	83 (59%)	33 (43%)	42 (91%)	8 (44%)	<0.001
Large cribriform	38 (27%)	6 (8%)	28 (61%)	4 (22%)	<0.001
Intraductal carcinoma	48 (34%)	21 (28%)	23 (50%)	4 (22%)	0.02
Gleason pattern 5					
Single cells and/or cords	99 (71%)	72 (95%)	10 (22%)	17 (94%)	<0.001
Small solid nests	23 (16%)	16 (21%)	1 (2%)	6 (33%)	0.003
Medium to large solid fields	15 (11%)	4 (5%)	5 (11%)	6 (33%)	0.006
Comedonecrosis	9 (6%)	2 (3%)	7 (15%)	0	0.02
Positive surgical margins	68 (49%)	40 (53%)	20 (44%)	8 (44%)	0.58
Pelvic lymph node dissection	91 (65%)	47 (62%)	29 (63%)	15 (83%)	0.22
Lymph node metastasis	12 (13%)	5 (11%)	4 (14%)	3 (20%)	0.54
Biochemical recurrence	68 (49%)	31 (41%)	29 (63%)	8 (44%)	0.05
Metastasis	36 (26%)	14 (18%)	18 (39%)	4 (22%)	0.04
Disease-specific death	12 (9%)	4 (5%)	7 (15%)	1 (6%)	0.17

Values denote either mean (median; IQR) or *n* (%).

*PSA* prostate-specific antigen.

**Fig. 2:**
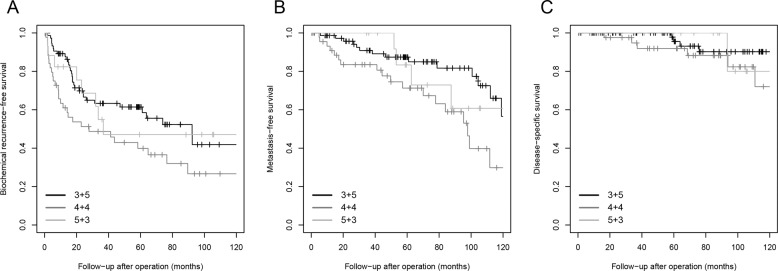
Survival curves stratified for individual Gleason score. Kaplan–Meier curves of **a** biochemical recurrence-free survival (log rank *P* = 0.001), **b** metastasis-free survival (log rank *P* < 0.001), and **c** disease-specific survival (log rank *P* = 0.01) in Gleason score 8 patients stratified for individual Gleason score.

Biochemical recurrence and postoperative distant metastasis were observed in 68 (49%) and 36 (26%) patients, respectively. Twenty-nine (63%) men with Gleason score 4 + 4 tumors experienced biochemical recurrence compared to 31 (41%, *P* = 0.02) with Gleason score 3 + 5 and 8 (44%, *P* = 0.78) with 5 + 3. Biochemical recurrence-free survival was significantly shorter for patients with Gleason score 4 + 4 than Gleason score 3 + 5 (log rank *P* = 0.02) prostate cancer. Gleason score 5 + 3 had the lowest absolute number of events and did not significantly differ from Gleason score 3 + 5 (log rank *P* = 0.82) and Gleason score 4 + 4 (log rank *P* = 0.26, Fig. 2). A similar trend was found for postoperative metastasis. Metastases occurred in 18 (39%) men with Gleason score 4 + 4 compared with 14 (18%, *P* = 0.01) with Gleason score 3 + 5 and 4 (22%, *P* = 0.20) men with Gleason score 5 + 3. Metastasis-free survival was significantly shorter for patients with Gleason score 4 + 4 than Gleason score 3 + 5 (log rank *P* = 0.006) prostate cancer. Gleason score 5 + 3 did not significantly differ from Gleason score 3 + 5 (log rank *P* = 0.63) and Gleason score 4 + 4 (log rank *P* = 0.25). The number of disease-specific deaths (*n* = 12, 9%) was too low for subgroup analysis.

### Cribriform architecture in Gleason score 8 prostate cancer

Invasive and/or intraductal cribriform carcinoma was present in 87 (62%) men, of whom 36 (41%) had Gleason score 3 + 5, 43 (49%) Gleason score 4 + 4, and 8 (10%) Gleason score 5 + 3 tumors. Of these, 83 (95%) had invasive and 48 (55%) had intraductal cribriform carcinoma. Both patterns were concurrently present in 44 (51%) men. Invasive cribriform carcinoma only was seen in 39 (44%) men and intraductal cribriform carcinoma only in 4 (5%) men. Large cribriform carcinoma was present in 37 (43%) men with cribriform architecture and was always accompanied by small cribriform carcinoma. Invasive and/or intraductal cribriform carcinoma was observed more frequently in Gleason score 4 + 4 than in Gleason score 3 + 5 (93% versus 47%, *P* < 0.001) and 5 + 3 (93% versus 44%, *P* < 0.001) tumors. Large invasive cribriform carcinoma also occurred more often in Gleason score 4 + 4 than in 3 + 5 (61% versus 8%, *P* < 0.001) or 5 + 3 (61% versus 22%, *P* < 0.001) tumors, while its appearance in Gleason score 3 + 5 and 5 + 3 was not significantly different (*P* = 0.08) in this cohort.

**Fig. 3:**
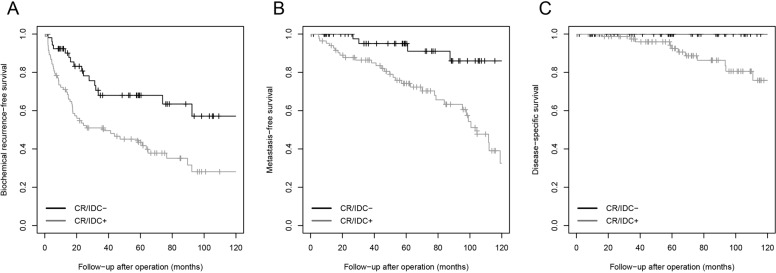
Survival curves stratified for cribriform architecture. Kaplan–Meier curves of **a** biochemical recurrence-free survival (log rank *P* = 0.001), **b** metastasis-free survival (log rank *P* < 0.001), and **c** disease-specific survival (log rank *P* = 0.01) in Gleason score 8 patients with invasive and/or intraductal cribriform carcinoma (CR/IDC+) and without invasive and/or intraductal cribriform carcinoma (CR/IDC−).

Gleason score 8 prostate cancer was stratified based on presence of invasive and/or intraductal cribriform carcinoma (Table 2). Non-organ confined disease (63% versus 34%, ≥pT3a, *P* = 0.003) and positive pelvic lymph nodes (19% versus 3%, *P* = 0.05) were more common in patients with cribriform architecture. Age, PSA levels, and surgical margin status were not significantly different between Gleason score 8 patients with or without cribriform architecture. Patients with cribriform architecture had significantly shorter biochemical recurrence-free (log rank *P* = 0.001), metastasis-free (log rank *P* < 0.001), and disease specific (log rank *P* = 0.01) survival than those without (Fig. 3).

**Table 2:** Gleason score 8 patients stratified for presence of cribriform architecture.

	Cribriform negative *n* = 53	Cribriform positive *n* = 87	*P* value
Age (years)	63.7 (64.1; 59.7–67.4)	65.4 (66.1; 62.7–69.2)	0.09
PSA (ng/ml)	12.5 (10.0; 7.0–14.0)	13.2 (10.0; 7.5–16.0)	0.33
pT-stage			
T2	35 (66%)	32 (37%)	0.003
T3a	10 (19%)	34 (39%)	
T3b/T4	8 (15%)	21 (24%)	
Gleason pattern 4	45 (85%)	86 (99%)	<0.001
Small cribriform	0	83 (95%)	<0.001
Large cribriform	0	38 (44%)	<0.001
Intraductal carcinoma	0	48 (55%)	<0.001
Gleason pattern 5	50 (94%)	60 (69%)	<0.001
Single cells and/or cords	48 (91%)	51 (59%)	<0.001
Small solid nests	10 (19%)	13 (15%)	0.54
Medium to large solid fields	0	15 (17%)	<0.001
Comedonecrosis	0	9 (10%)	0.02
Positive surgical margins	25 (47%)	43 (49%)	0.80
Pelvic lymph node dissection	32 (60%)	59 (68%)	0.37
Lymph node metastasis	1 (3%)	11 (19%)	0.05
Biochemical recurrence	16 (30%)	52 (60%)	0.001
Metastasis	4 (8%)	32 (37%)	<0.001
Disease-specific death	0	12 (14%)	0.004

Values denote either mean (median; IQR) or *n* (%).

*PSA* prostate-specific antigen.

### Histomorphology of Gleason pattern 5

Gleason pattern 5 was observed in 110 (79%) Gleason score 8 tumors. In addition to men with Gleason score 3 + 5 and 5 + 3, 16 (35%) men with Gleason score 4 + 4 had tertiary Gleason pattern 5. Single cells and/or cords were present in 99/110 (90%) and solid fields in 32/110 (29%) tumors. All Gleason 5 patterns were simultaneously present in 26 (24%) cases. Invasive and/or intraductal cribriform carcinoma was present in 23/32 (72%) cases with solid pattern 5 and in 36/78 (46%) cases with nonsolid pattern 5 (*P* = 0.02). Of interest, the nine solid field cases without associated cribriform architecture all were of the small nested type (Fig. 1d). Comedonecrosis was present in nine cases, 7 (78%) of which were present in Gleason score 4 + 4 tumors. Comedonecrosis was accompanied by cribriform architecture in all 9/9 (100%) cases and by solid fields in 5/9 (56%) cases. Moreover, comedonecrosis was observed more often in patients with large cribriform fields (7/20, 35%) than in those without (2/90, 2%, *P* < 0.001).

### Multivariable analysis of clinical outcome in Gleason score 8 patients

In univariate Cox regression analysis, pT3a (hazard ratio (HR) 2.1, 95% confidence interval (CI) 1.1–3.8, *P* = 0.02), pT3b/4 (HR 4.6, 95% CI 2.5–8.5, *P* < 0.001), Gleason score 4 + 4 (HR 1.9, 95% CI 1.1–3.1, *P* = 0.02), positive lymph nodes at time of operation (HR 11.8, 95% CI 5.6–25.2, *P* < 0.001), and overall presence of invasive and/or intraductal cribriform carcinoma (HR 2.4, 95% CI 1.4–4.1, *P* = 0.003) were significantly associated with shorter biochemical recurrence-free survival, while age (*P* = 0.18), PSA level (*P* = 0.43), Gleason score 5 + 3 (*P* = 0.78) and surgical margin status (*P* = 0.21) were not (Table 3). In multivariable analysis, pT3b/4-stage (HR 4.4, 95% CI 2.1–9.3, *P* < 0.001), positive lymph nodes (HR 9.9, 95% CI 4.2–23.5, *P* < 0.001), and overall cribriform architecture (HR 2.0, 95% CI 1.0–3.7, *P* = 0.04) had independent predictive value for biochemical recurrence-free survival, while Gleason score 4 + 4 (HR 1.7, 95% CI 1.0–2.9, *P* = 0.07) did not meet conventional measures of significance in this cohort. In case individual cribriform growth patterns were included in multivariable analysis instead of overall cribriform architecture, large invasive cribriform carcinoma (HR 2.0, 95% CI 1.0–4.1, *P* = 0.05) had independent predictive value for biochemical recurrence-free survival, whereas intraductal cribriform carcinoma (HR 1.3, 95% CI 0.8–3.5, *P* = 0.4) and small invasive cribriform carcinoma (HR 1.6, 95% CI 0.8–3.5, *P* = 0.2) did not (data not shown*)*.

**Table 3:** Cox regression analysis for biochemical recurrence-free survival in Gleason score 8 patients.

	Univariate			Multivariable		
	HR^a^	95% CI	*P* value	HR	95% CI	*P* value
Age (years)	1.0	0.9–1.0	0.18	1.0	0.9–1.0	0.05
PSA (ng/ml)	1.0	1.0–1.0	0.43	1.0	1.0–1.0	0.23
pT-stage						
T2	*ref*			*ref*		
T3a	2.1	1.1–3.8	0.02	1.8	1.0–3.4	0.06
T3b/T4	4.6	2.5–8.5	<0.001	4.4	2.1–9.3	<0.001
Gleason score						
3 + 5	*ref*			*ref*		
4 + 4	1.9	1.1–3.1	0.02	1.7	1.0–2.9	0.07
5 + 3	1.1	0.5–2.4	0.78	1.5	0.6–3.6	0.37
Positive surgical margins	1.4	0.8–2.2	0.21	0.8	0.5–1.5	0.55
Pelvic lymph node metastasis	11.8	5.6–25.2	<0.001	9.9	4.2–23.5	<0.001
Cribriform architecture	2.4	1.4–4.1	0.003	2.0	1.0–3.7	0.04

*CI* confidence interval.

^a^HR = hazard ratio.

Similar trends were observed for metastasis as pT3a (HR 2.7, 95% CI 1.2–6.2, *P* = 0.02), Gleason score 4 + 4 (HR 3.8, 95% CI 1.8–8.1, *P* = 0.001), positive lymph nodes (HR 11.5, 95% CI 5.2–25.9, *P* < 0.001), and overall cribriform architecture (HR 6.7, 95% CI 2.0–21.9, *P* = 0.002) were significantly associated with shorter metastasis-free survival, whereas age (*P* = 0.80), PSA level (*P* = 0.96), pT3b/4 (*P* = 0.06), Gleason score 5 + 3 (*P* = 0.85), and positive surgical margins (*P* = 0.95) were not (Table 4). In multivariable analysis, Gleason score 4 + 4 (HR 2.4, 95% CI 1.0–5.9, *P* = 0.05), positive lymph nodes (HR 15.0, 95% CI 5.6–40.0, *P* < 0.001), and overall cribriform architecture (HR 3.5, 95% CI 1.0–12.3, *P* = 0.05) had independent predictive value for metastasis-free survival. Due to the low number of events and risk of model overfitting we were not able to include individual cribriform or Gleason 5 growth patterns in multivariable analysis.

**Table 4:** Cox regression analysis for metastasis-free survival in Gleason score 8 patients.

	Univariate			Multivariable		
	HR^a^	95% CI	*P* value	HR	95% CI	*P* value
Age (years)	1.0	0.9–1.1	0.80	1.0	0.9–1.1	0.52
PSA (ng/ml)	1.0	1.0–1.0	0.96	1.0	0.9–1.0	0.50
pT-stage						
T2	*ref*			*ref*		
T3a	2.7	1.2–6.2	0.02	2.5	1.0–6.7	0.06
T3b/T4	2.4	1.0–6.0	0.06	1.3	0.4–3.8	0.67
Gleason score						
3 + 5	*ref*			*ref*		
4 + 4	3.8	1.8–8.1	0.001	2.4	1.0–5.9	0.05
5 + 3	1.1	0.32–4.0	0.85	0.7	0.2–3.0	0.65
Positive surgical margins	1.0	0.5–2.0	0.95	1.1	0.5–2.4	0.84
Pelvic lymph node metastasis	11.5	5.2–25.9	<0.001	15.0	5.6–40.0	<0.001
Cribriform architecture	6.7	2.0–21.9	0.002	3.5	1.0–12.3	0.05

*CI* confidence interval.

^a^HR = hazard ratio.

## Discussion

Our study demonstrates that among Gleason score 8 prostate cancer patients on radical prostatectomy, biochemical recurrence and metastases occur more often in Gleason score 4 + 4 than in Gleason score 3 + 5 or 5 + 3 tumors. Invasive and/or intraductal cribriform carcinoma was observed in 62% of tumors and was associated with adverse pathological features and clinical outcome. Cribriform architecture occurred more frequently in men with Gleason score 4 + 4 (93%) than in those with Gleason score 3 + 5 (47%) and 5 + 3 (44%). In multivariable analysis, cribriform architecture was an independent parameter for biochemical recurrence- and metastasis-free survival, while Gleason score was not. Therefore, cribriform architecture also has important value for risk stratification among Gleason score 8 prostate cancer patients.

Since the introduction of Grade Groups several reports have analyzed the clinical outcome of Gleason score 3 + 5, 4 + 4, and 5 + 3 prostate cancer [4]. Some of these studies found that men with Gleason score 3 + 5 at radical prostatectomy had reduced risk of biochemical recurrence among Gleason score 8 patients [23, 24]. Others did not find a difference among Gleason score 8 subgroups or concluded that men with primary Gleason pattern 5 had worse outcome [16, 25–28]. This variability of results might be explained by the use of different specimen types and clinical outcome measures [29]. Furthermore, Gleason score 8 is relatively uncommon, hampering statistical analysis on large numbers of patients or resulting in clustering of Gleason score 5 + 3 and 5 + 3 tumors [28, 30]. Finally, from a morphologically point of view, Gleason score 8 prostate cancer is a very heterogeneous disease including highly variable quantities of Gleason 3, 4, and 5 growth patterns. This heterogeneity might lead to significant interobserver variability in tumor grading. For instance, Shah et al. only found fair interobserver reproducibility for Gleason pattern 5 assignment among 16 international expert genitourinary pathologists [31]. Upon rereview of 40 archival cases with Gleason score 5 + 3 prostate cancer, Kryvenko et al. assigned the same score in only 4 (10%) specimens, but upgraded 57.5% and downgraded 17.5% of cases [32].

Many studies demonstrated worse clinical outcome for patients with cribriform architecture [7–11]. Most of these studies investigated cribriform architecture in intermediate grade prostate cancer, while the impact of cribriform architecture in Gleason score ≥4 + 3 is less well established. In Gleason score 7–10 prostate cancer biopsy patients, presence of cribriform architecture has been associated with advanced pathological stage and worse disease-specific survival compared with those with cribriform-negative biopsies [14, 33]. Harding-Jackson et al. found cribriform architecture, but not Gleason score, to have independent predictive value for cancer-specific survival in Gleason score 8 patients [16]. In the current study, we confirmed that cribriform architecture had strong discriminative value, even in aggressive Gleason score 8 prostate cancer. Both overall invasive cribriform and/or intraductal carcinoma as well as its more aggressive large cribriform variant were significantly more often observed in Gleason score 4 + 4, than 3 + 5 and 5 + 3 disease. Since its association with adverse outcome, the high frequency of cribriform architecture might well explain the worse outcome of Gleason score 4 + 4 prostate cancer compared with those with 3 + 5 in our study, while the low number of 5 + 3 patients hampered powerful statistical analysis. Of interest, however, is that in multivariable analysis not only cribriform architecture but also Gleason score 4 + 4 had independent prognostic value for metastasis-free survival. A similar trend was observed for biochemical recurrence-free survival although the predictive value of Gleason score 4 + 4 did not reach conventional measures of significance (*P* = 0.07). This implicates that other grading factors apart from cribriform architecture contribute to the worse outcome in Gleason score 4 + 4 patients. A possible explanation could be that Gleason score 4 + 4 disease has the lowest percent of Gleason 3 growth pattern, which is by definition present in less than 5% of the tumor volume. In 3 + 5 disease, percent Gleason pattern 3 theoretically varies from 50% to 95%, while it occupies 5% to 50% in Gleason score 5 + 3 tumors. Some groups have shown independent prognostic value for percent Gleason pattern 4 and 5, which outperformed Gleason score [34]. The inverse could well be true for percent Gleason pattern 3; if a tumor still has the biological capacity to mature into well-delineated glandular structures it is associated with better outcome.

Little is known about the predictive value of individual Gleason grade 5 growth patterns, which have been reported as either single cells, cords, small solid cylinders, solid fields, and presence of comedonecrosis [3]. Single cells and/or cords are the most common Gleason pattern 5 [35, 36]. Flood et al. found that presence of solid fields and number of different Gleason 5 growth patterns were associated with shorter biochemical recurrence-free survival in Gleason score 9–10 prostatectomies [35]. Compared with other Gleason 5 patterns, comedonecrosis was associated with non-organ confined disease and biochemical recurrence [19, 37]. While individual Gleason 4 growth patterns have increasingly been subject to clinicopathological analysis, information on the clinical relevance of Gleason 5 patterns is still scarce. Our group recently performed in-depth three-dimensional visualization of prostate adenocarcinoma architectural growth patterns and revealed two separate morphological groups [38]. The first group consists of a tubular network in which the vast majority if not all tumor cells are in direct contact with surrounding stroma. This group encompasses the morphological continuum of Gleason pattern 3, poorly formed and fused pattern 4, and single cells and cords pattern 5. The second group has contiguous epithelial proliferations in which the majority of tumor cells are not in contact with surrounding stroma and consists of cribriform pattern 4 and solid pattern 5 with or without comedonecrosis. Our current finding that solid fields mostly coexisted with cribriform structures is reflective of this continuum. In the current study, we distinguished between small nested cylinders consisting up to 30 tumor cells and larger solid fields. While the latter was continuous with cribriform growth, small nested cylinders were not. This suggests that both have different biological and possibly clinical relevance. However, larger studies are required to perform statistical analysis on the clinical relevance of individual Gleason 5 growth patterns.

Strong points of this study are the detailed histological review of radical prostatectomy specimens and the classification of cribriform architecture with the use of strict morphological criteria and additional immunohistochemistry. The study is limited by the retrospective study design. The inclusion of high-grade samples from two participating centers could have resulted in a selection bias. Furthermore, the relatively short follow-up of 59 months and limited number of patients restricted robust statistical analysis.

In conclusion, Gleason score 8 is a heterogeneous group of prostate cancers. Although clinicopathological characteristics of Gleason score 3 + 5, 4 + 4, and 5 + 3 are mostly similar, Gleason score 4 + 4 patients have a higher risk of adverse events. Cribriform architecture is an independent predictor for metastasis-free survival and has better discriminative value for clinicopathological outcome than Gleason score. Therefore, reporting cribriform architecture might add value in risk stratification of Gleason score 8 prostate cancer patients.
